# Transcriptomic insights into the molecular regulatory mechanism of staminody in Chinese narcissus (*Narcissus tazetta* subsp. *chinensis*)

**DOI:** 10.3389/fpls.2026.1931382

**Published:** 2026-09-18

**Authors:** Pengda Zhao, Yuting Yang, Na Zhang, Keke Fan, Yanting Chang, Baokun Yan, Hanchen Tian, Zehui Jiang, Yanjun Ma, Tao Hu

**Affiliations:** 1 International Center for Bamboo and Rattan, Beijing, China; 2 Key Laboratory of National Forestry and Grassland Administration/Beijing for Bamboo & Rattan Science and Technology, Beijing, China

**Keywords:** *Narcissus tazetta*, protein interaction, staminody, TGA, transcriptional complex

## Abstract

Staminody (petaloid stamen) contributes to floral diversity in horticultural plants, yet its molecular regulation remains poorly understood. Using two Chinese narcissus varieties, ‘Jinzhanyintai’ (single-flowered) and ‘Yulinglong’ (double-flowered), we conducted anatomical and histological analyses, which confirmed that the petaloid stamens of ‘Yulinglong’ arise from a homeotic transformation of normal stamens of ‘Jinzhanyintai’, as evidenced by conserved internal locule-like structures and gradual morphological intermediates. Comparative transcriptomics across five key developmental stages revealed global gene expression dynamics during staminody. Screening for differentially expressed transcription factors across these stages identified a bZIP TGA-subfamily homolog, designated *NtTGA10*, which was consistently and significantly downregulated in petaloid stamens compared with normal stamens. Subcellular localization confirmed nuclear enrichment of NtTGA10. Bimolecular fluorescence complementation, luciferase complementation imaging, and co-immunoprecipitation assays demonstrated that NtTGA10 specifically interacts *in vivo* with the C-class MADS-box protein NtMADS6 (AGAMOUS), suggesting formation of a transcriptional complex. RT-qPCR analysis showed high expression of *NtTGA10* in normal stamens but significant downregulation in petaloid stamens and other floral organs. This expression pattern implies that *NtTGA10* not only participates in normal stamen development but also its downregulation may be closely associated with petaloid conversion. Collectively, our findings suggest that the NtTGA10–NtMADS6 transcriptional complex serves as a critical regulator in maintaining stamen identity and repressing petaloid conversion. This study provides the first evidence for TGA–MADS box interplay in regulating floral organ identity in narcissus, offering novel insights into the molecular basis of floral organ plasticity and identifying potential targets for molecular design breeding in horticultural plants.

## Introduction

Plant morphogenesis is a precisely regulated and orderly developmental process (Frohlich, 2006). A flower is the key reproductive and ornamental organ of plants, and therefore its development has always been a major focus in plant biology; also, the diversity of floral forms holds significant ornamental and economic value (Frohlich, 2006). Among various floral variant types, double flowers have garnered widespread attention due to their complex structure and diverse morphology (Luo et al., 2024).

A typical flower comprises four concentric whorls of organs, from the outside inward: sepals, petals, stamens, and carpels. Single flowers have only one whorl of petals, whereas double flowers exhibit multiple layers of petaloid structures (Jiang et al., 2023). The core distinction lies in organ identity: double flowering often involves the transformation of reproductive organs, such as stamens or carpels, into petaloid structures, a phenomenon termed “petalody” or “staminody” (Zhao and Liu, 2009). Staminody, also referred to as homeotic conversion of stamens into petaloid organs (Ronse De Craene, 2007), is one of the key mechanisms underlying double-flower formation (Xiong, 2019), which can be classified as partial or complete based on the degree of transformation (Liu et al., 2019). When stamens undergo complete petaloid conversion but carpels do not, male-sterile materials can be generated (Jiang et al., 2023). This process is specifically characterized by the disappearance of anthers and the flattening and expansion of stamens, culminating in petal-like structures (He, 2017). This represents a frequent developmental phenomenon in ornamental species, widely observed in various plants such as *Eriobotrya japonica* and *Hippeastrum hybridum (*He, 2017; Ou et al., 2023).

The genetic regulation of floral organ identity primarily follows the classic ABC model. This model posits that the ectopic or extended expression of genes belonging to classes A, B, or C can induce the petaloid conversion of floral organs (Pelaz et al., 2000). Specifically, class A genes not only are involved in sepal and petal development but also antagonize class C genes to co-regulate petal and stamen numbers (Bowman et al., 1991). The key causes for double-flower formation include loss-of-function mutations in class C genes or overexpression and spatial expansion of the expression domains of class B genes; furthermore, the expression of class C genes is itself repressed by class A genes (Bowman et al., 1991). Subsequently proposed models, such as the ABCDE model (Kim et al., 2005) and the quartet model (Theißen and Saedler, 2001), have further refined the regulatory network of floral organ development based on the ABC framework (Qi et al., 2024). *AGAMOUS* (*AG*), a class C gene within the MIKC-type MADS-box family, not only controls the differentiation and development of stamens and carpels but also regulates floral meristem termination (Yanofsky et al., 1990; Sieburth et al., 1995). Its loss of function has been confirmed to cause double-flower phenotypes in various plants including *Arabidopsis thaliana (*Yanofsky et al., 1990) and morning glory (Sage-Ono et al., 2011), as well as in monocotyledonous ornamentals such as lily, where downregulation of the *AG* homolog *LelAG* is closely associated with increased staminody in double-flowered cultivars (Akita et al., 2011).

However, the transcriptional co-factors that modulate *AG* function or act downstream of *AG* in stamen development remain largely unexplored. Recent studies in *Arabidopsis* have implicated bZIP transcription factors of the TGA subfamily in anther development (Murmu et al., 2010; Sun, 2020). Among these, the TGA gene family belongs to the D subfamily of bZIP transcription factors, with members *TGA9* and *TGA10* playing crucial roles in floral organogenesis (Sun, 2020). In *A. thaliana*, *TGA9* and *TGA10* share high sequence similarity and overlapping expression patterns. The *tga9/tga10* double mutant exhibits defects in anther development similar to those of the *roxy1/roxy2* double mutant. Previous studies have indicated that TGA9/10 can directly interact with ROXY1/2, with glutaredoxins regulating the transcriptional activity of TGA9/10 during this process (Murmu et al., 2010). These findings suggest that TGA transcription factors may be evolutionarily conserved in maintaining stamen identity across different species. In tobacco, silencing *TGA2.1* similarly induces the formation of petaloid stamens (Thurow et al., 2005).

The cultivated varieties of Chinese narcissus (*Narcissus tazetta* subsp. *chinensis*) provide an ideal system for investigating the aforementioned phenomenon. The single-flowered cultivar ‘Jinzhanyintai’ possesses the typical floral structure, whereas the double-flowered cultivar ‘Yulinglong’ exhibits complete stamen petalody (**Figures 1B, D**). Comparative analyses of karyotypes (Zhuang et al., 1999), genetic diversity (Chen et al., 2002), and chloroplast genome sequencing (Long et al., 2021) all indicate an extremely close phylogenetic relationship between the two. Importantly, mutant individuals of ‘Jinzhanyintai’ with incomplete staminody and inflorescences bearing both single and double flowers have been occasionally observed in the field (**Figures 1A, C**). Therefore, ‘Yulinglong’ is widely regarded as a natural staminody mutant of ‘Jinzhanyintai’ (Ma et al., 2023). However, the continuous anatomical characteristics of its petaloid conversion process have not been systematically depicted. Also, the key molecular regulators driving this identity switch, along with their mechanisms of action, remain unknown.

**Figure 1:**
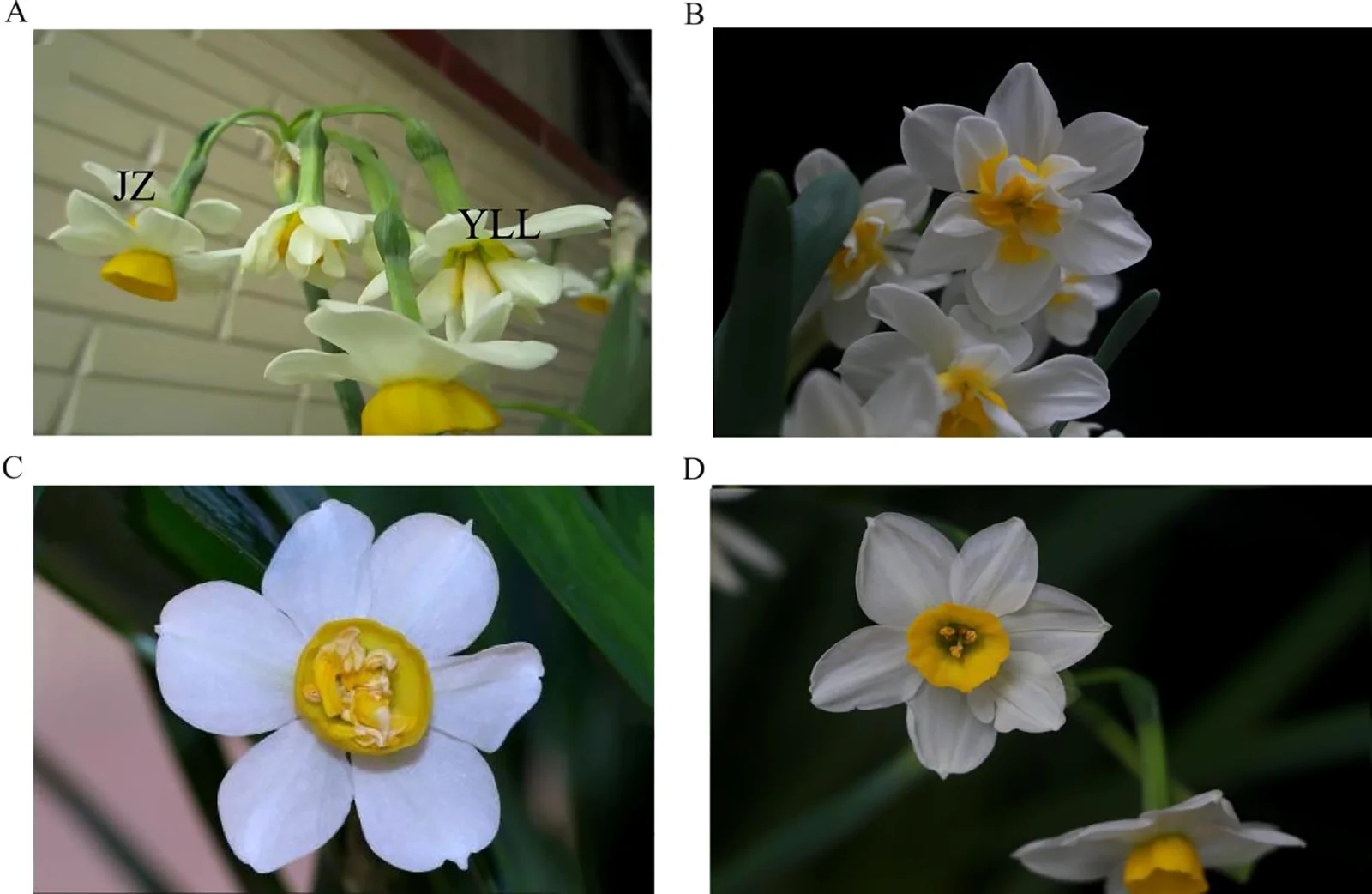
Flower morphology of Chinese narcissus. **(A)** Co-occurrence of ‘Jinzhanyintai’ and ‘Yulinglong’ flower types on the same inflorescence. **(B)** ‘Yulinglong’ (double-flowered). **(C)** An incompletely petaloid intermediate staminody mutant. **(D)** ‘Jinzhanyintai’ (single-flowered).

In this study, we aimed to elucidate the morphological dynamics and molecular regulators underlying staminody in Chinese narcissus. By integrating comparative transcriptomics, expression profiling, and protein–protein interaction analyses, we identified a TGA transcription factor, NtTGA10, that interacts with the C-class MADS-box protein NtMADS6, suggesting a novel regulatory module governing stamen identity in this horticulturally important species.

## Materials and methods

### Plant materials

Flower buds and the largest florets within the inflorescences from three-year-old commercial bulbs of ‘Jinzhanyintai’ (JZ) and ‘Yulinglong’ (YLL) were used as plant materials. Both cultivars were produced in Zhangzhou, Fujian Province, China. Healthy bulbs were selected in early June according to the standard LY/T 1633-2005, transported to Beijing (39jingrted’i’’d 116jingrted’i’’d) in late July, and temporarily stored in a shaded, ventilated environment. Temperature and humidity during storage were monitored using a COS-03-X recorder (Shandong Renke Measurement and Control Technology Co., Ltd., Jinan, China). During storage, the average daily relative humidity was maintained above 51%, the daily maximum temperature was kept below 31 °C, and the daily minimum temperature ranged between 21 °C and 24 °C. From July 31 to late October, flower buds from the main bulbs and the largest florets within the inflorescences were sampled at regular intervals (approximately every 6 days during early and middle stages, and at longer intervals during later stages) according to the key morphological changes defined in the Results; the five key stages selected for transcriptome analysis were stages 4, 6, 8, 10, and 14. Additionally, *Nicotiana benthamiana* and *Arabidopsis thaliana* plants used in this study were cultivated in controlled growth chambers under a 14-h light/10-h dark photoperiod (20 °C/16 °C) with 70% relative humidity. All samples were flash-frozen in liquid nitrogen and stored at –80 °C for subsequent analysis (Ma et al., 2023).

### Morphological observation

Inflorescences and individual florets were manually dissected using needles or forceps under a stereomicroscope (Stemi 305, Carl Zeiss AG, Germany) to examine floral organ development, with particular emphasis on the staminody process. Images were captured using an Axiocam ERc 5s camera (ZEISS, Germany). At least three individual flowers from each developmental stage and each cultivar were observed.

### Paraffin sectioning

The largest florets from early inflorescence spathes and from middle-to-late stage inflorescences were collected and fixed in Formalin-Acetic acid-Alcohol (FAA) fixative (Cat No.: G1108, 50% ethanol). Paraffin sectioning was performed as follows: (1) Dehydration and clearing: tissues were dehydrated through an ethanol series (75% for 4 h, 85% for 2 h, 90% for 2 h, 95% for 1 h, and absolute ethanol twice for 30 min each), followed by clearing in ethanol–benzene and xylene (Sinopharm, catalog #10023418) for 5–10 min each. (2) Infiltration and embedding: tissues were infiltrated with molten paraffin at 65 °C three times (1 h each) and embedded using a JB-P5 embedding machine (model JB-L5, Wuhan Junjie Electronic Co., Ltd.). Blocks were solidified on a –20 °C freezing stage (JB-L5). (3) Sectioning and mounting: trimmed blocks were cooled and sectioned into 4-µm slices using an RM2016 microtome (Leica Biosystems, Germany). Sections were flattened on a 40 °C water bath using a KD-P tissue flotation bath (Jinhua Kedi Instrument Equipment Co., Ltd.) and then dried at 60 °C. (4) Dewaxing and rehydration: sections were treated with eco-friendly dewaxing clearing solution (Cat No.: G1128) twice for 20 min each, followed by rehydration through absolute and graded ethanol. (5) Staining: sections were stained with safranin staining solution (Cat No.: G1031) for 2 h, rinsed with tap water, destained through graded ethanol, and counterstained with fast green for 6–20 s. (6) Clearing and mounting: after clearing in xylene, sections were mounted with neutral balsam (10004160). (7) Observation and imaging: sections were observed under a Primo Star microscope (Carl Zeiss AG, Germany), and images were acquired using an Axiocam 208 color camera (ZEISS, Germany). At least three individual flowers from each stage and each cultivar were sectioned and observed.

### Scanning electron microscope

SEM sample preparation was conducted as follows: (1) Sampling and primary fixation: fresh tissue samples (am mm²) were excised within 1–3 min, avoiding stretching or compression, briefly rinsed with PBS, and immediately immersed in electron microscopy fixative (2.5% glutaraldehyde in 0.1 M phosphate buffer, pH 7.4). Samples were fixed at room temperature for 2 h and then stored at 4 °C. For adherent cells, culture medium was removed, cells were gently rinsed with PBS, and fixation was performed using the same protocol. (2) Post-fixation: fixative was removed, and samples were washed three times with 0.1 M phosphate buffer (PB, pH 7.4), 15 min per wash. Samples were then post-fixed in 1% osmium tetroxide (OsO_4_) prepared in 0.1 M PB for 1–2 h at room temperature in the dark, followed by three washes with 0.1 M PB (pH 7.4), 15 min per wash. (3) Dehydration: samples were dehydrated through a graded ethanol series (30%, 50%, 70%, 80%, 90%, 95%, 100% and 100%) for 15 min per step, followed by immersion in isoamyl acetate for 15 min. (4) Drying: dehydrated samples were dried using a critical point dryer (CPD). (5) Conductive coating: dried samples were mounted on conductive carbon-tape-coated stubs and sputter-coated with gold–palladium for approximately 30 s using an ion sputter coater. (6) SEM imaging: prepared samples were examined and imaged using a scanning electron microscope. For pre-dried or inorganic samples, the dehydration and CPD steps were omitted. Three biological replicates per stage and per cultivar were examined.

### Transcriptome sequencing and analysis

#### RNA extraction, library construction, and sequencing

Samples of normal stamens from JZ and petaloid stamens from YLL were collected at five developmental stages (4, 6, 8, 10, and 14). Total RNA was extracted using the Tiangen polysaccharide and polyphenol plant total RNA extraction kit (DP441, Tiangen Biotech, Beijing, China). RNA integrity was assessed using an Agilent 2100 Bioanalyzer (Agilent Technologies, Santa Clara, CA, USA). Libraries were constructed using the Hieff Next−Generation Sequencing (NGS) Ultima Dual−mode mRNA Library Prep Kit (12309ES, Yeasen Biotechnology, Shanghai, China). The procedure included mRNA enrichment, fragmentation, double−stranded cDNA synthesis, end repair, adapter ligation, and PCR amplification. Paired−end 150−bp sequencing was performed on an Illumina NovaSeq 6000 platform (Illumina, San Diego, CA, USA). This study employed second−generation transcriptome sequencing technology and used the third−generation full−length transcripts previously constructed by our research group as the reference transcriptome(https://www.omicsmart.com/index.php) (Ma et al., 2023). Library construction and sequencing were performed by Guangzhou Gidio Biotechnology Co., Ltd. (Guangzhou, Guangdong, China).

#### Data quality control

Raw sequencing reads were subjected to quality control using fastp software to remove adapter sequences, reads containing an excessive proportion of N bases, and low-quality reads, thereby yielding high-quality clean reads. Subsequently, base composition and quality score distribution of the clean reads were examined to ensure that the data met the requirements for subsequent analysis. The clean reads were aligned to the reference transcriptome of *N. tazetta* using HISAT2 (v2.2.0) with default parameters. Gene expression levels were quantified as FPKM (Fragments Per Kilobase of transcript per Million mapped reads) using StringTie (v2.1.4).

#### Differential gene expression analysis

Based on the gene expression count matrix, differential expression analysis was performed using DESeq2 software (v1.34.0). The screening threshold was set at |log_2_Fold Change| ≥ 1 and adjusted *P*-value (padj) < 0.05. For each developmental stage comparison between JZ and YLL, volcano plots were generated to visualize differential gene expression. Significant DEGs were subjected to expression pattern clustering and visualized as heatmaps using pheatmap software.

#### Functional enrichment analysis

DEGs common across multiple developmental stages were identified. A Venn diagram was drawn using the VennDiagram tool to extract the core gene set, which was then subjected to GO and KEGG enrichment analyses. The transcription factors among the DEGs were systematically identified by aligning the protein sequences of all DEGs against the Plant Transcription Factor Database (PlantTFDB) using BLASTP (*E*-value ≤ 1e-5). All genes annotated as transcription factors based on the database’s family annotation were selected. Further statistical analysis was performed according to the PlantTFDB classification system for in-depth exploration.

### RT-qPCR validation

Nine differentially expressed genes were selected for validation of the RNA-sequencing data by RT-qPCR, with *NtActin* as the internal reference. Gene-specific primers were designed using Primer Premier 5.0 (Premier Biosoft, Palo Alto, CA, USA) (**Supplementary Table 1**). RT-qPCR reactions were performed on a LightCycler 480 system using TB Green Premix Ex Taq II (RR820A; TaKaRa Bio, Shiga, Japan) following the program described previously (Zhang et al., 2025). Relative expression levels were calculated using the 2^-ΔΔCt^ method. Three biological replicates were used per sample, each with three technical replicates. Pearson correlation analysis was performed using GraphPad Prism 9.5 (GraphPad Software, San Diego, CA, USA) to evaluate the consistency between RT-qPCR and RNA-seq data.

### *NtTGA10* gene cloning and sequence analysis

The full-length coding DNA sequence (CDS) of *NtTGA10* was amplified via high-fidelity PCR using 2× Phanta Max Master Mix (P515; Vazyme Biotech, Nanjing, Jiangsu, China) with specific primers (**Supplementary Table 1**) designed based on its coding sequence, using cDNA from JZ stamens as the template. The amplified PCR product was cloned into the pMD19-T vector (TaKaRa, Japan) and sequenced for verification. After cloning and sequence verification, similarity analysis was performed using NCBI BLAST, and a phylogenetic tree was constructed using MEGA 11 software (Fan et al., 2022).

### Subcellular localization

The constructed *NtTGA10*-EGFP fusion expression vector was transformed into *Agrobacterium tumefaciens* strain GV3101 via heat shock. Positive clones were selected and cultured. The bacterial pellet was resuspended in induction medium containing acetosyringone to an OD_600_ of approximately 1.0, followed by activation at room temperature. The suspension was then injected into *N. benthamiana* leaves. The infiltrated plants were kept in the dark for 12 h and then transferred to a normal light cycle for 48 h. Samples were taken for DAPI staining, and co-localization of DAPI and GFP signals was observed under a confocal microscope (Carl Zeiss AG, Germany) (Fan et al., 2022; Hu et al., 2023).

### Protein–protein interaction validation

To investigate the regulatory mechanism of NtTGA10 in staminody, we turned to the classic ABCDE model, in which AG-class genes are central determinants of stamen and carpel identity and their loss of function or downregulation has been widely shown to cause stamen petaloidy. Based on this, together with our previous identification of NtMADS6, an AGAMOUS (AG)-like MADS-box gene belonging to the C-class floral organ identity genes, we hypothesized that NtTGA10 may function through forming a transcriptional complex with an AG-class protein. To test this hypothesis, we selected NtMADS6 and NtTGA10 for protein–protein interaction assays.

#### Bimolecular fluorescence complementation

The interaction between NtTGA10 and NtMADS6 was validated via BiFC analysis using an *Agrobacterium* GV3101-mediated transient expression system in *N. benthamiana*. The experimental group comprised *Agrobacterium* mixtures co-expressing YCE-NtMADS6 and YNE-NtTGA10. As a positive control, we used a vector pair known to reconstitute YFP fluorescence (YNE + YCE, which served as a reference for system validity). Negative controls were YNE-NtTGA10 + YCE and YNE + YCE-NtMADS6. The mixtures were infiltrated into the epidermis of *N. benthamiana* leaves. Plants were kept in the dark for 24 h and then transferred to light for an additional 48 h before fluorescence signals were observed under a laser scanning confocal microscope (Carl Zeiss AG, Germany) (Zhang et al., 2025).

#### LCI assay

A dual-luciferase reporter assay was conducted using an *Agrobacterium*-mediated transient expression system in *N. benthamiana*. The constructed reporter plasmids, NtMADS6-nLuc and *NtTGA10*-cLuc, were separately transformed into *A. tumefaciens* strain GV3101. The experimental setups were as follows: the experimental group co-expressed NtMADS6-nLuc and *NtTGA10*-cLuc; As a negative control, the empty nLuc and cLuc vectors were co-expressed; and the negative controls were NtMADS6-nLuc + cLuc and *NtTGA10*-cLuc + nLuc.

Transformed *Agrobacterium* was cultured at 30 °C for 2 days. Single colonies were inoculated into 5 mL of LB liquid medium and shaken at 28 °C and 220 rpm overnight. Cells were pelleted by centrifugation at 4000 rpm for 4 min, the supernatant was discarded, and the pellet was resuspended in infiltration buffer (containing 10 mM MgCl_2_, 10 mM MES, and 20 µM acetosyringone) to an OD_600_ of approximately 0.8. The suspension was activated by incubation at 28 °C for 2 h. The activated *Agrobacterium* mixtures were combined in a 1:1 ratio according to the experimental design and infiltrated into *N. benthamiana* leaves using a needleless 1-mL syringe. Injected plants were grown under low light for 2 days before *in vivo* imaging or sample collection (Yi, 2021).

#### Co-immunoprecipitation

The protein interaction between NtTGA10 and NtMADS6 was validated via Co-IP using an *Agrobacterium*-mediated transient expression system in *N. benthamiana*. The constructed expression vectors 35S:NtTGA10-FLAG and 35S:NtMADS6-MYC were electroporated into *A. tumefaciens* GV3101 competent cells. *Agrobacterium* harboring these vectors or the control 35S::GFP-MYC were separately activated, resuspended in infiltration buffer to an OD_600_ of 0.6, and allowed to stand at room temperature for 4 h. The mixtures of NtTGA10-FLAG and NtMADS6-MYC strains (1:1 ratio) were infiltrated into *N. benthamiana* leaves, with a mixture of NtTGA10-FLAG and GFP-MYC serving as the negative control. Leaf samples were collected 3 days post-infiltration and flash-frozen in liquid nitrogen.

Co-IP was performed at 4 °C. Total protein was extracted using chilled IP lysis buffer, vortexed, incubated on ice for 20 min, and centrifuged at 13,000×g for 10 min at 4 °C. A portion of the supernatant was mixed with five times the loading buffer and boiled as the input. The remaining supernatant was incubated with FLAG magnetic beads for 2 h. The beads were washed five times with phosphate-buffered saline, and the bound proteins were eluted by boiling in 2× loading buffer. The eluted samples and the input were analyzed by SDS-PAGE and Western blot (Shen et al., 2024; Han et al., 2025).

### Real-time quantitative PCR

To investigate the potential role of *NtTGA10* in stamen development, its expression pattern was examined in normal stamens of JZ, petaloid stamens of YLL, and other floral tissues (tepals, corona, gynoecium, hypanthium, bracts, and leaves) by RT-qPCR. Gene-specific primers were designed using Primer Premier 6 (**Supplementary Table 1**), with *NtActin* as the internal reference as described previously (Shen et al., 2024). RT-qPCR was performed on a LightCycler 480 system using TB Green Premix Ex Taq II (RR820A; TaKaRa Bio, Shiga, Japan). Relative expression levels were calculated using the 2^−ΔΔCt^ method (Wang, 2018; Livak and Schmittgen, 2001). Statistical significance among tissues was determined by one-way ANOVA followed by Tukey’s HSD post-hoc test (SPSS version 25, IBM, Armonk, NY, USA), and data were visualized using GraphPad Prism.

## Results

### Anatomical and histological analysis

Chinese narcissus has a cymose inflorescence, each containing 3–7 florets arranged in whorls. Starting from the initiation of the first-whorl perianth primordia (August 1), the floral organogenesis of JZ and YLL was observed and recorded under a stereomicroscope. The floral bud development of the two cultivars was highly similar (**Figure 2A**): both possessed white, semi-translucent spathes that enclosed the inflorescence in a flattened, ovoid shape. As the florets gradually increased in size and number, and the inflorescence axis elongated, the flower buds became longer and wider.

**Figure 2:**
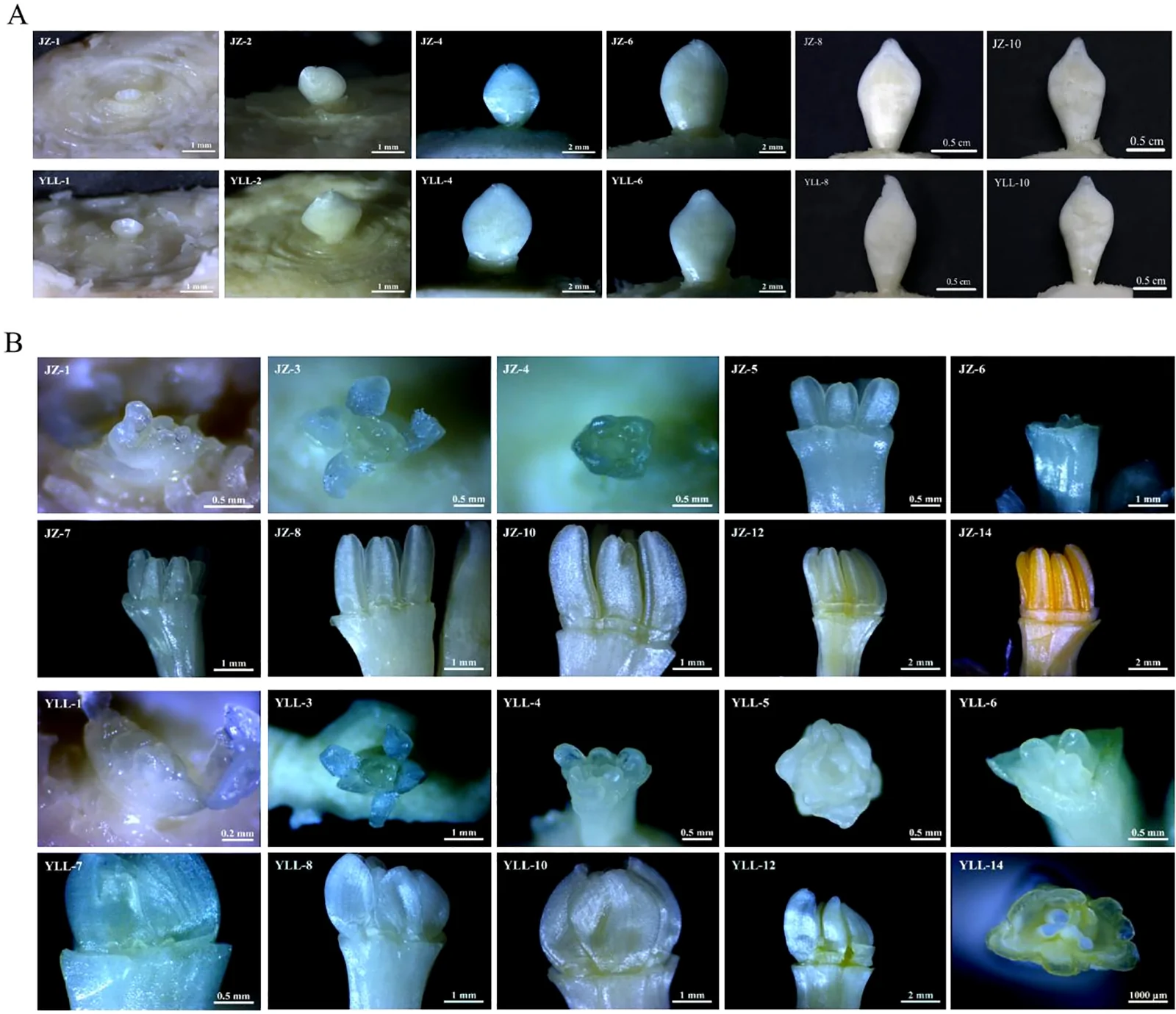
Floral bud and floral organ morphology of JZ and YLL at different developmental stages. **(A)** Flower buds pictures of JZ and YLL in different period. **(B)** Floral organ pictures of JZ and YLL in different periods. JZ, ‘Jinzhanyintai’; YLL, ‘Yulinglong’; number in the picture, different periods. JZ-4, without first whorl tepals; JZ-5, JZ-7~JZ-14, YLL-4~YLL-5, YLL-7~YLL-12, without tepals; JZ-6, without tepals and stamens; YLL-6 and YLL-14, without tepals and petalized stamens.

Stages 1 (July 31) and 2 (August 6): perianth primordium differentiation. As shown in **Figure 2B** (JZ-1 and YLL-1), the bracts of both cultivars had already differentiated by stage 1. After removing the bracts, the first-whorl perianth primordia of the first floret on the inflorescence were visible as protrusions. Six days later (stage 2), the first-whorl perianth primordia of JZ-2 and YLL-2 had further differentiated; both cultivars had developed second-whorl perianth primordia, and first-whorl stamen primordia were clearly visible. The differentiation of second-whorl perianth primordia occurred almost simultaneously with that of first-whorl stamen primordia.

Stage 3 (August 12): second-whorl stamen primordia emerged in JZ-3 and YLL-3, while second-whorl perianth primordia gradually differentiated. At this stage, stamen primordia of the two cultivars were identical in morphology, appearing as elliptical protrusions with smooth surfaces.

Stage 4 (August 18): a three-lobed stigma was clearly visible, indicating that the gynoecium primordia had differentiated in both cultivars. At this stage, the two cultivars began to diverge: stamen primordia of JZ-4 remained elliptical, with first-whorl stamens starting to elongate and a longitudinal groove appearing in the middle of the adaxial side; in contrast, the six stamen primordia of YLL-4 exhibited an inward “cat-ear”-like folding.

Stages 5 (August 24) and 6 (August 30): the hypanthium above the ovary began to elongate. The gynoecium of YLL-6 developed significantly faster than that of JZ-6, and stamen primordia had become completely petaloid. In JZ-6, the stamens were nearly fully formed: six anthers each showed three longitudinal grooves on the adaxial side, and filaments started to elongate; anthers could be easily detached using dissecting forceps.

Stage 7 (September 5): conical protrusions appeared at the base of the third- and fourth-whorl petaloid stamens of YLL. Stage 8 (September 11): irregular lamellar protrusions, distributed as discontinuous spots, emerged at the base of the third- and fourth-whorl petaloid stamens; meanwhile, petaloid stamens folded inward to form a semi-open barrel-shaped structure. Stage 9 (September 17): both petaloid stamens of YLL and normal stamens of JZ continued to enlarge. By stage 14 (October 17), JZ stamens had fully matured and turned yellow, whereas YLL petaloid stamens had completed petaloid transformation.

As shown in **Figure 2**, stamen primordia of YLL were morphologically highly similar to those of JZ in early stages, both being elliptical, and the arrangement of the six primordia was also identical: first-whorl stamen (or petaloid stamen) primordia were alternate with first-whorl tepals, and second-whorl stamen (or petaloid stamen) primordia were alternate with second-whorl tepals. Stamen petalody in YLL initiated at stage 4, when stamen primordia began to develop an inward-folding “cat-ear”-like morphology. At the same stage, stamen primordia of JZ began to differentiate a central longitudinal groove. By stage 6, petalody of YLL stamens was nearly complete, whereas the external morphology of JZ stamens was largely established but not yet mature.

Later-stage anatomical investigations revealed transitional forms (**Figure 1C**). Mature stamens of JZ (**Figure 3A**, a) displayed the typical structure with four anther locules. In contrast, a typical petaloid stamen of YLL (**Figure 3A**, b) exhibited a bi-lobed, petal-like structure. Intermediate, incompletely petaloid stamens were observed in some YLL samples (e.g., stages 8 and 14) (**Figures 3A**, c, d). These structures retained a stamen-like base with color changes but exhibited abnormal anther locule morphology. Removal of the petaloid structure revealed a degenerated filament (**Figure 3A**, e).

**Figure 3:**
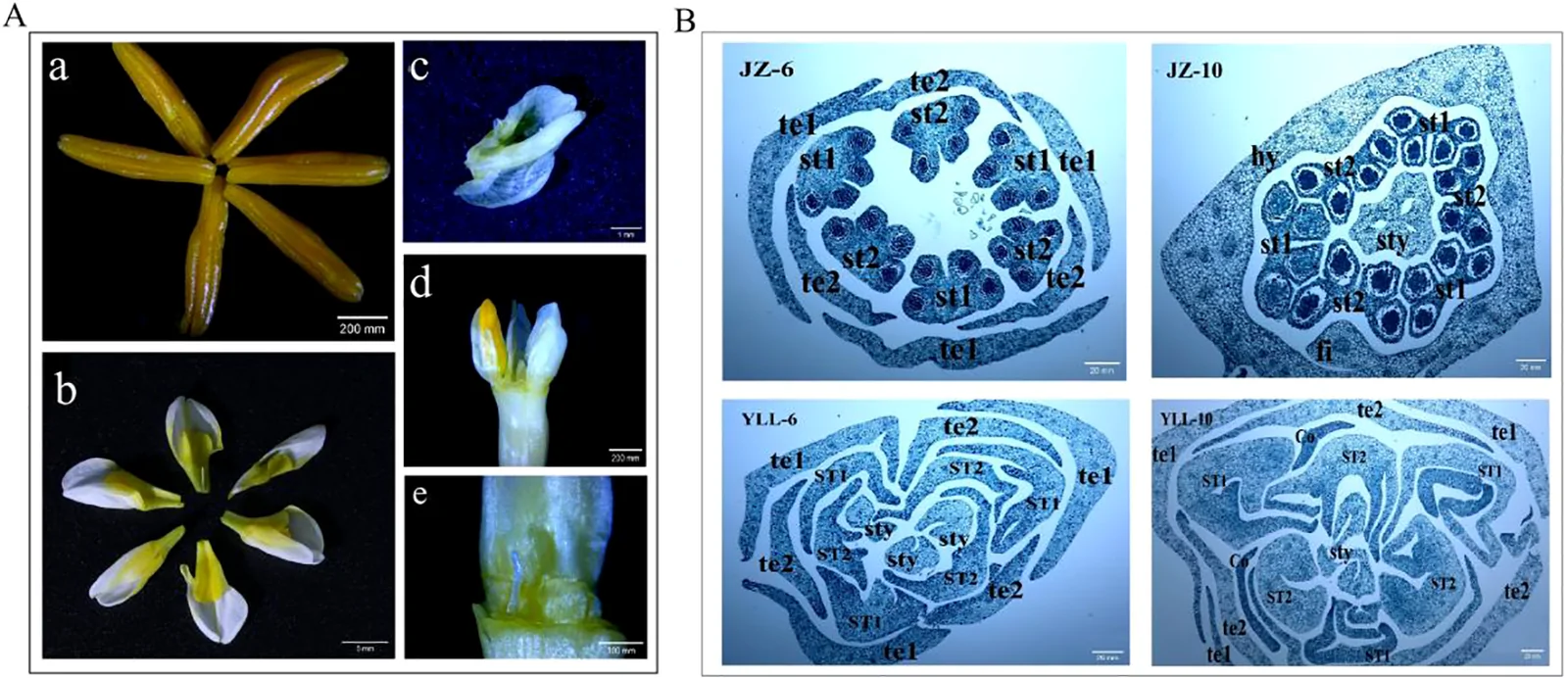
Anatomical diagram of the stamen petalody process. **(A)** Abnormal petalody pictures of YLL stamen. **(a)** JZ-st: Normal stamen after morphogenesis in JZ. **(b)** YLL-ST: Petaloid stamen of ‘Yulinglong’ (YLL). **(c)** YLL-STa: An incompletely petaloid stamen found in YLL-8.d. YLL-STb: An incompletely petaloid stamen observed in YLL-14. **(e)** YLL-STc: A degenerated filament visible in YLL-14. **(B)** Transverse paraffin-embedded sections of stamens from JZ and petaloid stamens from YLL. The development of stamens and petaloid stamens was divided into 14 stages. Five representative stages (4, 6, 8, 10, and 14) were selected for observation. Abbreviations: st, stamen; te, tepal; ST, petaloid stamen; hy, hypanthium; Co, corona. Numbers 1 and 2 indicate the first and second whorls, respectively (e.g., te1: first-whorl tepal).

Transverse paraffin sectioning of JZ stamens and YLL petaloid stamens was performed to further examine these phenotypes at the histological level (**Figure 3B**). The results showed that each JZ stamen possessed four independent anther locules. Transverse sections of YLL petaloid stamens revealed a similar “quadripartite” developmental tendency, where the number of structural partitions corresponded precisely to the number of anther locules in JZ stamens.

High-resolution scanning electron microscopy (SEM) was employed to compare the morphology of stamens in single-flowered JZ and petaloid stamens in double-flowered YLL at stages 8 and 12.

At stage 8, JZ stamen primordia exhibited a swollen, globose structure with smooth, undifferentiated epidermal cells (**Figure 4A**). In contrast, YLL stamen primordia showed pronounced ectopic flattening and significant expansion in the proximodistal axis, with a wavy surface contour (**Figure 4C**).

**Figure 4:**
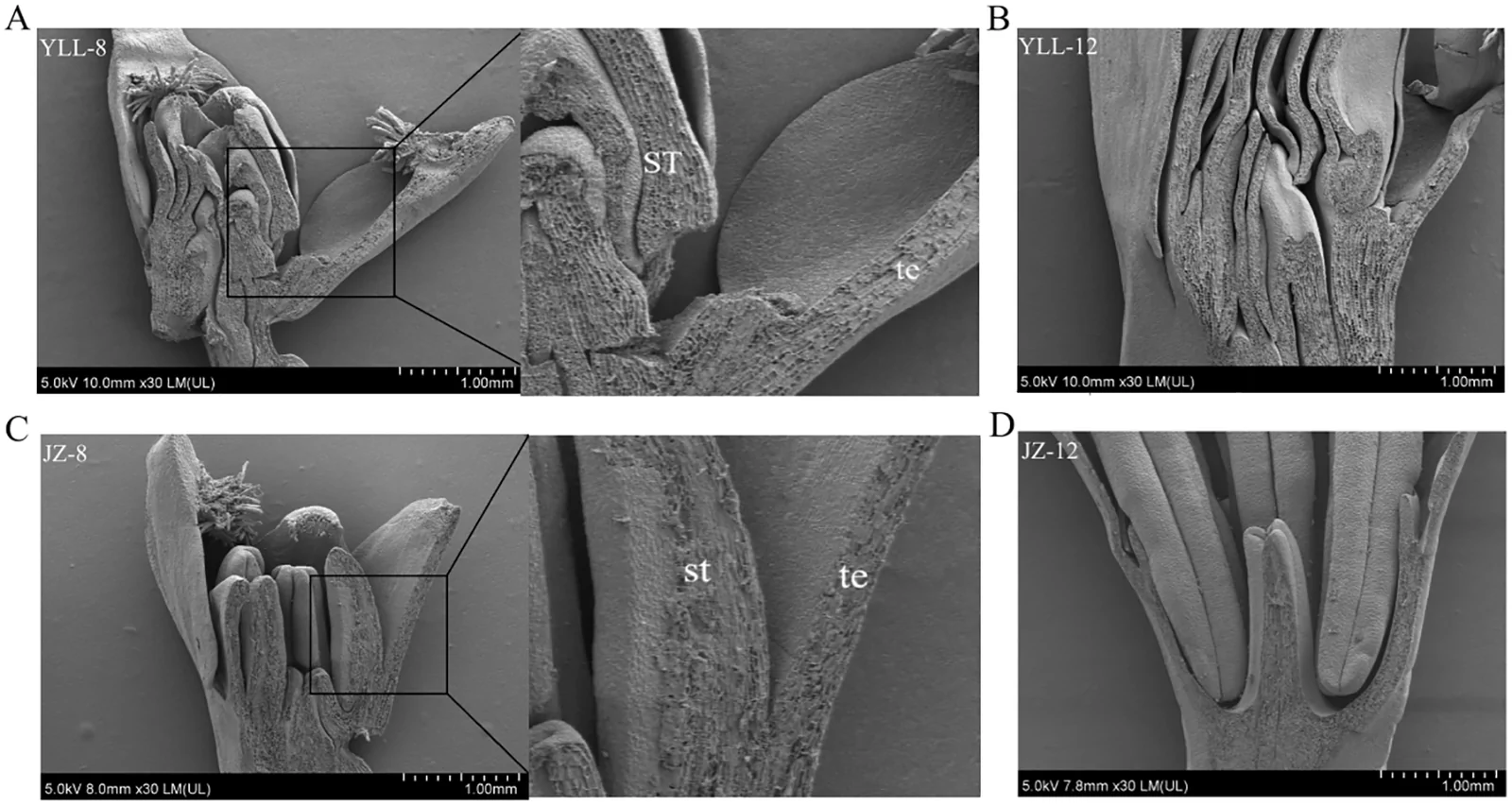
Scanning electron microscopy observation of key stages of normal stamen (JZ) and petaloid stamen (YLL) development. **(A)** Scanning electron micrograph and magnified view of stage VIII of normal stamen development in JZ. **(B)** Scanning electron micrograph and magnified view of stage XII of normal stamen development in JZ. **(C)** Scanning electron micrograph and magnified view of stage VIII of petaloid stamen development in YLL. **(D)** Scanning electron micrograph and magnified view of stage XII of petaloid stamen development in YLL.

At stage 12, JZ stamens had completed development, displaying a bilobed anther structure, with each lobe containing two distinct microsporangia; the epidermal surface featured papillary cells and stomatal complexes (**Figure 4B**). In contrast, YLL third-whorl organs had transformed into broad, flattened, margin-undulated laminar tissues. High-magnification SEM revealed that epidermal cells of these ectopic organs exhibited jigsaw puzzle-shaped morphology, with large cells and sinuous anticlinal walls, rather than the small, uniform cells typical of anther epidermis. No pollen grains or stomatal structures were observed on their surface (**Figure 4D**).

### Quality assessment of transcriptome sequencing data

To elucidate the regulatory mechanisms underlying normal stamen development in JZ and petaloid stamen formation in YLL, normal stamens from JZ and petaloid stamens from YLL were collected at five developmental stages (4, 6, 8, 10, and 14). Total RNA was extracted and subjected to transcriptome sequencing. Raw sequencing reads were processed for quality control using fastp software to remove adapter sequences, reads containing excessive N bases, and low-quality reads, yielding high-quality clean reads. A total of 201,940,0316 clean reads were obtained, with Q30 > 92.8% and GC content averaging 45.77% across all samples (**Table 1**), confirming that the data quality met the requirements for subsequent analyses.

**Table 1:** Statistics of sequencing data quality assessment.

Sample	Raw reads	Clean reads	Bases numbers (Gb)	Q20 (%)	Q30 (%)	GC (%)
JZ-st-4-1	65993746	65826812	9.831	97.43	93.05	46.46
JZ-st-4-2	57663846	57502872	8.580	97.73	93.55	46.24
JZ-st-4-3	68467554	68274528	10.222	97.32	92.83	45.93
JZ-st-6-1	56946300	56813706	8.500	97.54	93.23	46.02
JZ-st-6-2	68456004	68285378	10.219	97.67	93.57	46.19
JZ-st-6-3	53615392	53482840	8.011	97.42	92.98	46.26
JZ-st-8-1	107833590	107627154	16.066	98.30	94.80	44.60
JZ-st-8-2	56214120	56073416	8.392	97.63	93.49	46.56
JZ-st-8-3	48240802	48095806	7.193	97.56	93.29	44.92
JZ-st-10-1	67343260	67189420	10.012	97.55	93.31	45.78
JZ-st-10-2	63203752	63078994	9.359	97.66	93.58	45.53
JZ-st-10-3	71428370	71240802	10.656	97.51	93.21	46.47
JZ-st-14-1	53547170	53450202	7.995	98.30	94.91	45.66
JZ-st-14-2	69662278	69539154	10.403	98.12	94.45	45.81
JZ-st-14-3	63493696	63387438	9.456	98.41	95.23	45.67
YLL-ST-4-1	59119930	58975756	8.795	98.02	94.32	47.15
YLL-ST-4-2	67920172	67820786	10.118	98.44	95.30	46.12
YLL-ST-4-3	56962106	56813584	8.479	97.79	93.66	46.63
YLL-ST-6-1	83994588	83847032	12.523	98.46	95.24	44.39
YLL-ST-6-2	61718274	61604582	9.213	98.03	94.26	45.51
YLL-ST-6-3	58331606	58125800	8.648	97.41	92.80	44.58
YLL-ST-8-1	69284510	69144560	10.340	98.21	94.76	45.65
YLL-ST-8-2	72855694	72719452	10.873	98.10	94.42	45.70
YLL-ST-8-3	78600390	78451864	11.694	98.25	94.86	45.44
YLL-ST-10-1	77945464	77802842	11.636	98.25	94.80	45.45
YLL-ST-10-2	71304620	71171028	10.647	98.37	95.13	45.89
YLL-ST-10-3	82876170	82742566	12.366	98.09	94.39	45.28
YLL-ST-14-1	70256872	70113352	10.479	98.31	94.95	45.39
YLL-ST-14-2	78304424	78151290	11.677	98.22	94.73	45.58
YLL-ST-14-3	62153718	62047300	9.272	98.29	94.89	46.25
Average	67457947.27	67313343.87	10.06	97.95	94.13	45.77
Sum	2023738418.00	2019400316.00	301.66			

### Differential gene expression analysis

Principal component analysis revealed high correlations among samples (**Figure 5A**). Genes associated with staminody were identified through pairwise comparisons between libraries from normal stamens and petaloid stamens across their respective developmental stages. Gene expression levels were quantified using the FPKM (Fragments Per Kilobase per Million mapped fragments) method.

**Figure 5:**
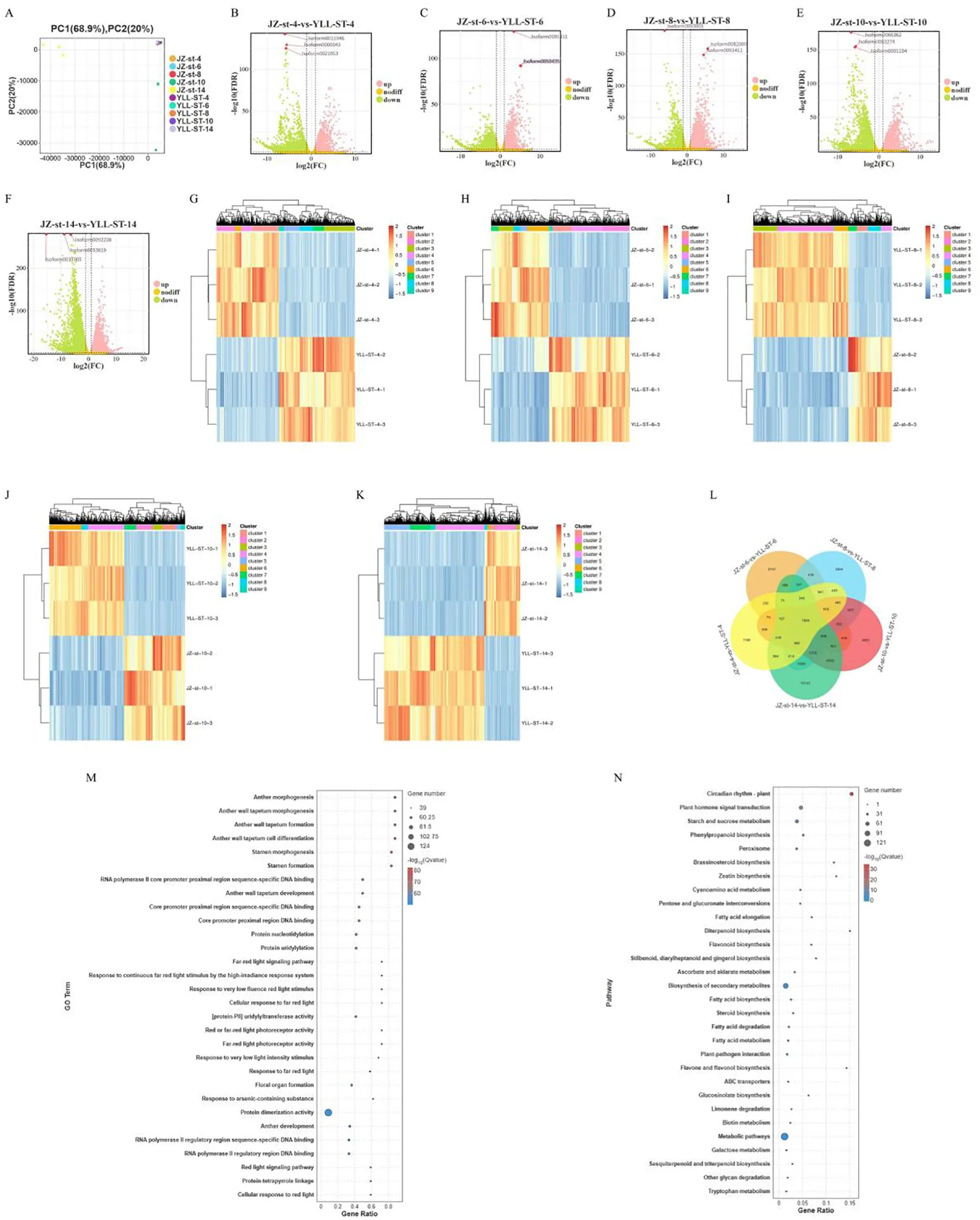
Differential gene expression analysis. **(A)** Principal component analysis. The x-axis represents the first principal component (PC1) and the y-axis represents the second principal component (PC2). **(B–F)** Volcano plots of DEGs between YLL and JZ in stages 4, 6, 8, 10, and 14, respectively. Pink dots represent upregulated genes, green dots represent downregulated genes, and yellow dots represent non-DEGs. **(G–K)** Heatmaps of DEGs between YLL and JZ in stages 4, 6, 8, 10, and 14, respectively. The top dendrogram shows single-gene clustering; shorter branch distances indicate more similar gene expression patterns. The left dendrogram shows sample clustering, with corresponding sample names on the right. **(L)** Venn diagram showing the overlap of DEGs identified from comparisons in stages 4, 6, 8, 10, and 14 between YLL and JZ. Colors represent: yellow (stage 4), orange (stage 6), light blue (stage 8), red (stage 10), and green (stage 14). **(M)** GO enrichment analysis. **(N)** KEGG pathway enrichment analysis. The x-axis represents the enrichment strength. The y-axis lists the GO terms/KEGG pathways, categorized as described by the legend (“Class”) on the right. Each dot represents a GO term/KEGG pathway; larger dots indicate a greater number of associated DEGs.

A total of 6,661 DEGs were identified in stage 4, comprising 3,656 upregulated and 3,005 downregulated genes (**Figure 5B**). In stage 6, 8,479 DEGs were obtained (4,931 upregulated and 3,548 downregulated; **Figure 5C**). Stage 8 yielded 13,468 DEGs (9,241 upregulated and 4,227 downregulated; **Figure 5D**). Stage 10 produced 17,347 DEGs (9,583 upregulated and 7,764 downregulated; **Figure 5E**). In stage 14, 22,978 DEGs were identified (16,953 upregulated and 6,025 downregulated; **Figure 5F**).

The log_10_(FPKM) values of the DEGs were used for normalization and clustering analysis. Hierarchical clustering analysis grouped the DEGs into nine clusters (**Figures 5G–K**). In stage 4, five clusters were upregulated and four downregulated (**Figure 5G**). In stage 6, four clusters were upregulated and five downregulated (**Figure 5H**). Stage 8 showed four upregulated and five downregulated clusters (**Figure 5I**). Stage 10 had six upregulated and three downregulated clusters (**Figure 5J**), whereas stage 14 showed four upregulated and five downregulated clusters (**Figure 5K**).

The Venn diagram (**Figure 5L**) showed the overlap of DEGs across the five comparative groups, revealing a core set of 1,425 common DEGs. These common DEGs were subjected to GO and KEGG pathway enrichment analyses. GO enrichment analysis (**Figure 5M**) showed significant enrichment in categories related to anther development, light signal response, adenosine triphosphate binding, and protein kinase activity. KEGG pathway analysis (**Figure 5N**) revealed significant enrichment in plant hormone signal transduction, secondary metabolite biosynthesis, and carbohydrate and fatty acid metabolism pathways.

### Screening for TFs associated with staminody

Because transcription factors orchestrate developmental transitions, we next focused our differential expression analysis on the transcription factor families represented among the DEGs. Systematic differential expression analysis was performed on the transcriptome data from normal stamens of JZ and petaloid stamens of YLL. A total of 231 transcription factors belonging to 25 gene families were identified through annotation and statistical analysis of DEGs. Among these, members of several TF families, including bHLH, ARF, AP2, and bZIP, exhibited significant changes in expression between the two materials (**Figure 6A**). Within the bZIP family, the TGA subfamily showed high expression in normal stamens and downregulated expression in petaloid stamens. Comparison of expression levels among TGA subfamily members revealed that *NtTGA10* was constitutively highly expressed across the key developmental stages of normal stamens, whereas its expression was extremely low in petaloid stamens (**Figure 6B**). Based on this differential expression pattern, *NtTGA10* was selected for further functional characterization.

**Figure 6:**
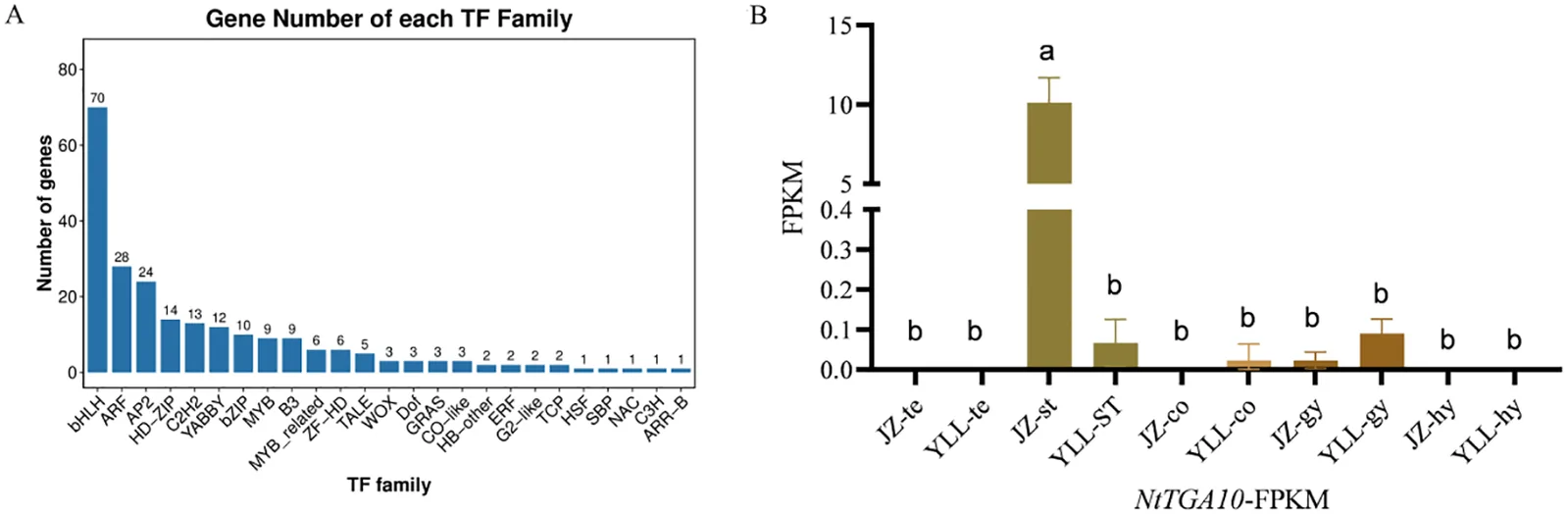
Transcription factor family statistics and expression profile of NtTGA10 in the transcriptome. **(A)** Statistics of TF families. **(B)** Expression pattern of *NtTGA10* in the transcriptome data. te: tepal; st: stamen; ST: petaloid stamen; co: corona; gy: gynoecium; hy: hypanthium. Letters “a” and “b” indicate statistically significant differences across various tissues (*p* < 0.05).

### Validation of RNA-seq data by qRT-PCR

To validate the reliability of the transcriptome data, nine significantly differentially expressed transcription factors were selected for expression validation using real-time quantitative PCR (RT-qPCR). The results showed that the qPCR expression trends of these genes were largely consistent with the RNA-Seq sequencing data (**Figure 7**), confirming the accuracy and reliability of the RNA-Seq data.

**Figure 7:**
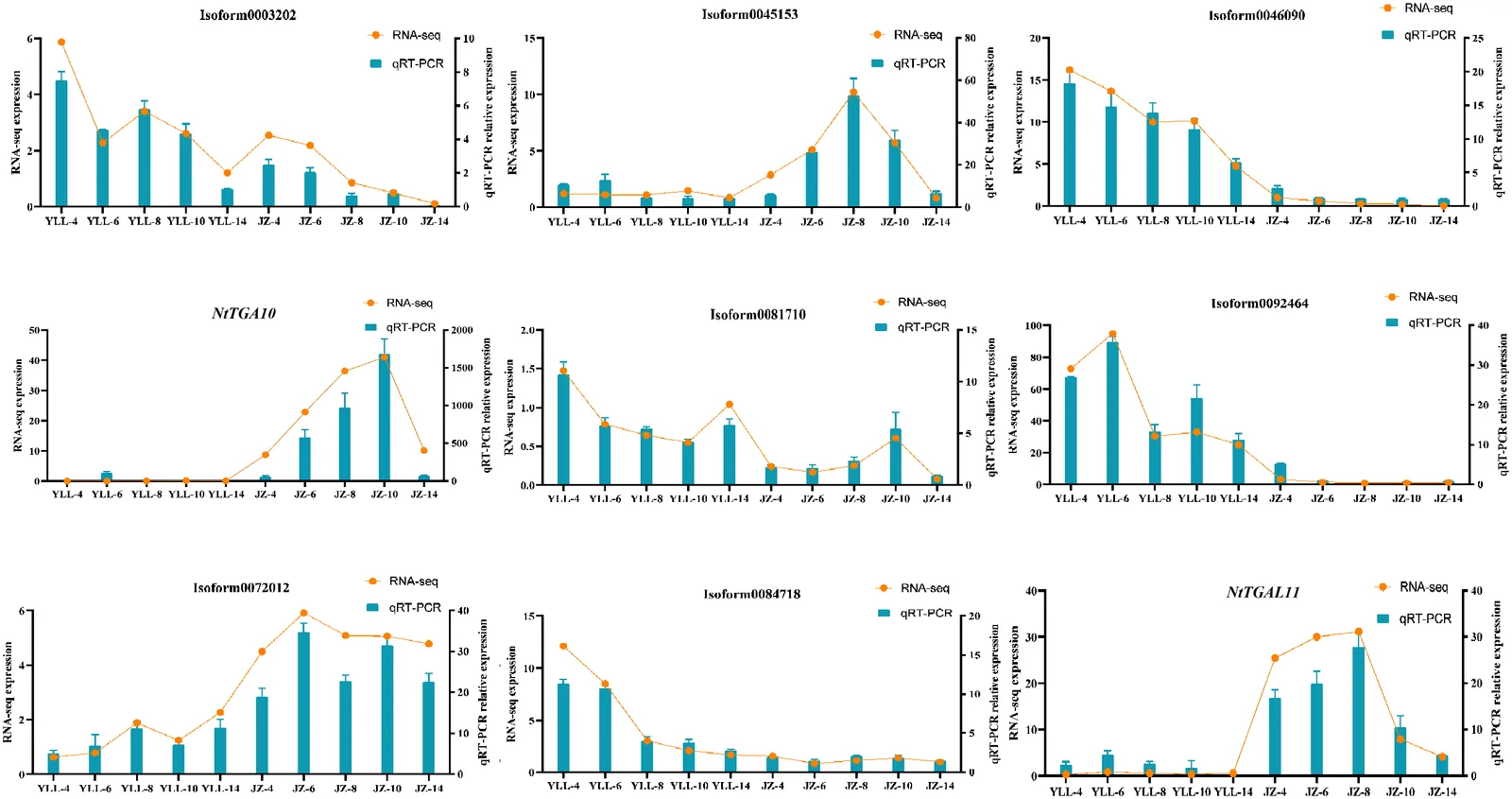
Validation of transcriptome data by qPCR. Relative expression levels of nine identified differentially expressed genes shown by qRT-PCR (bar chart, right Y-axis) and by FPKM values (line graph, left Y-axis). The *Ntactin* gene was used as the reference for calculating relative expression levels in both qRT-PCR and RNA-seq (FPKM values). Error bars indicate the standard deviation of three independent replicates in qRT-PCR.

### Cloning and multiple sequence alignment of NtTGA10

Basic Leucine Zipper (bZIP) transcription factors constitute a superfamily widely distributed in eukaryotes, and can be classified into ten subgroups (A–S) based on sequence variations in their structural domains. Among these, the D-subgroup TGA (TGACG-binding) factors are named for their specific recognition and binding to the TGACG core motif, and represent one of the most intensively studied subclades within the bZIP family. Numerous studies have demonstrated that bZIP transcription factors play pivotal regulatory roles in floral organ development. In this study, we focused on the TGA subfamily and systematically analyzed the expression patterns of its member genes during stamen petalody based on transcriptomic data. From this analysis, we identified *NtTGA10* and aimed to further investigate its biological function in staminody.

To examine the phylogenetic relationship of NtTGA10, a phylogenetic tree was constructed based on its amino acid sequence alongside 10 TGA proteins from *Arabidopsis thaliana*. Evolutionary analysis revealed that NtTGA10 was most closely related to AtTGA10 and AtTGA9 (**Figure 8A**). Multiple sequence alignment confirmed that NtTGA10 contained an N-terminal bZIP domain and a C-terminal DOG1 domain (**Figure 8B**).

**Figure 8:**
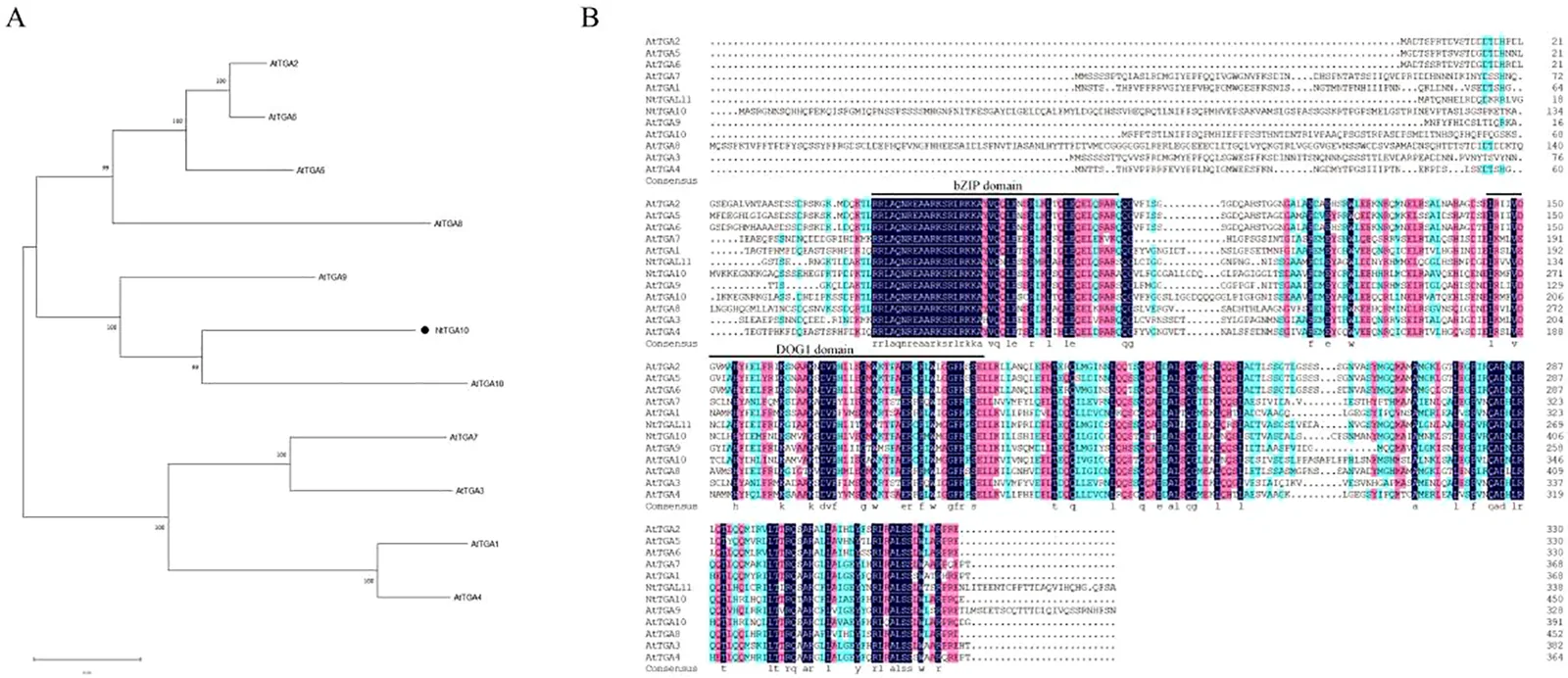
Phylogenetic analysis and multiple sequence alignment of NtTGA10 with Arabidopsis TGA family members. **(A)** Phylogenetic tree analysis of *NtTGA10* from ‘Jinzhanyintai’ and TGA subfamily proteins from *A. thaliana*. The tree includes 10 *Arabidopsis* TGA sequences, with the black dot representing NtTGA10. **(B)** Multiple sequence alignment of the ‘Jinzhanyintai’ NtTGA10 protein with *Arabidopsis* TGA amino acid sequences. Amino acid sequence similarity is color-coded: dark blue for 100%, pink for ≥75%, and light blue for ≥50%. Detailed sequence information is provided in the **Supplementary Information**.

### Subcellular localization of NtTGA10

To determine the subcellular localization of NtTGA10, 35S::EGFP (empty vector control) and 35S::NtTGA10-EGFP (fusion expression) vectors were constructed and transiently expressed in tobacco epidermal cells via *Agrobacterium*-mediated transformation. As shown in **Figure 9**, the EGFP fluorescence signal in the control group was distributed throughout the entire cell. In contrast, the fluorescence signal of the NtTGA10-EGFP fusion protein was predominantly enriched in the nucleus and showed substantial overlap with the nuclear dye DAPI (4′,6-diamidino-2-phenylindole).

**Figure 9:**
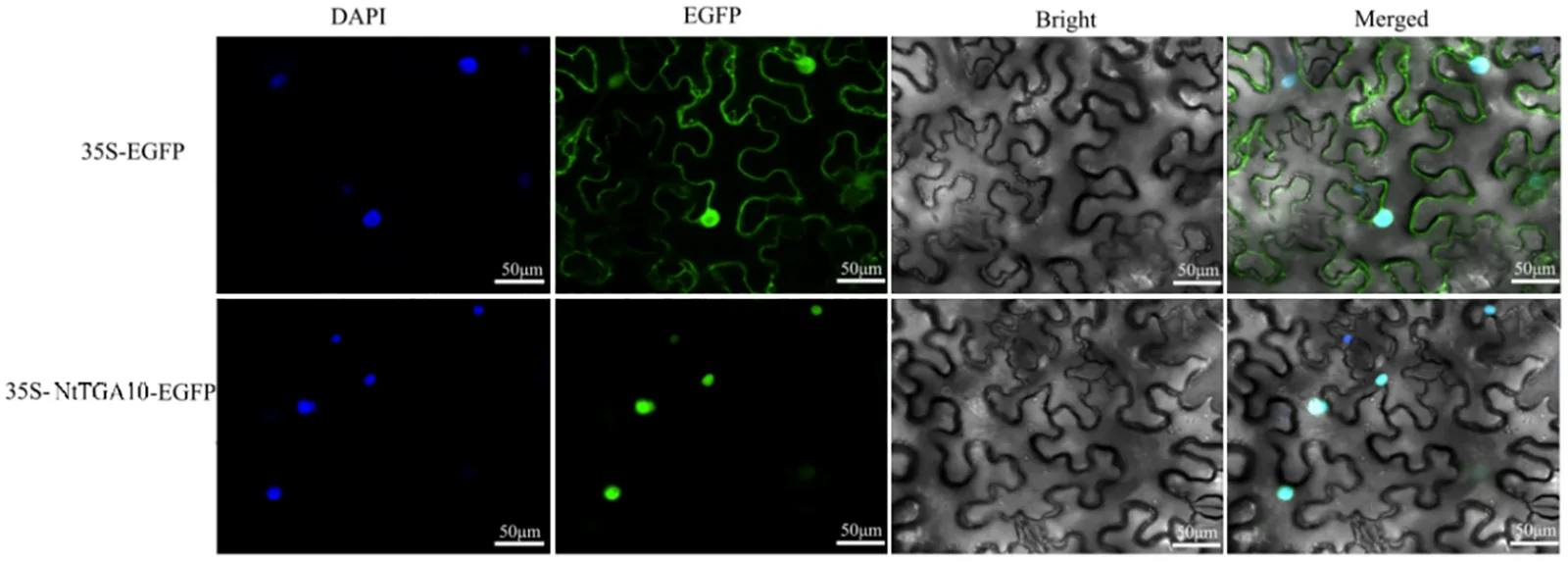
Subcellular localization of NtTGA10. DAPI: Nuclear staining with DAPI. 35S-EGFP: Fluorescence of EGFP protein driven by the 35S promoter. 35S::NtTGA10-EGFP: Fluorescence of the NtTGA10-EGFP fusion protein driven by the 35S promoter. The merged images show co-localization of the signals. All proteins were transiently expressed in tobacco leaves.

### Verification of protein–protein interaction between NtTGA10 and NtMADS6

The protein–protein interaction between NtTGA10 and the AG-class gene NtMADS6 was validated using BiFC, LCI, and Co-IP assays.

In the BiFC assay (**Figure 10A**), EGFP fluorescence signals were detected in both the cytoplasm and the nucleus, overlapping with DAPI staining. No significant fluorescence was observed in the control experiments.

**Figure 10:**
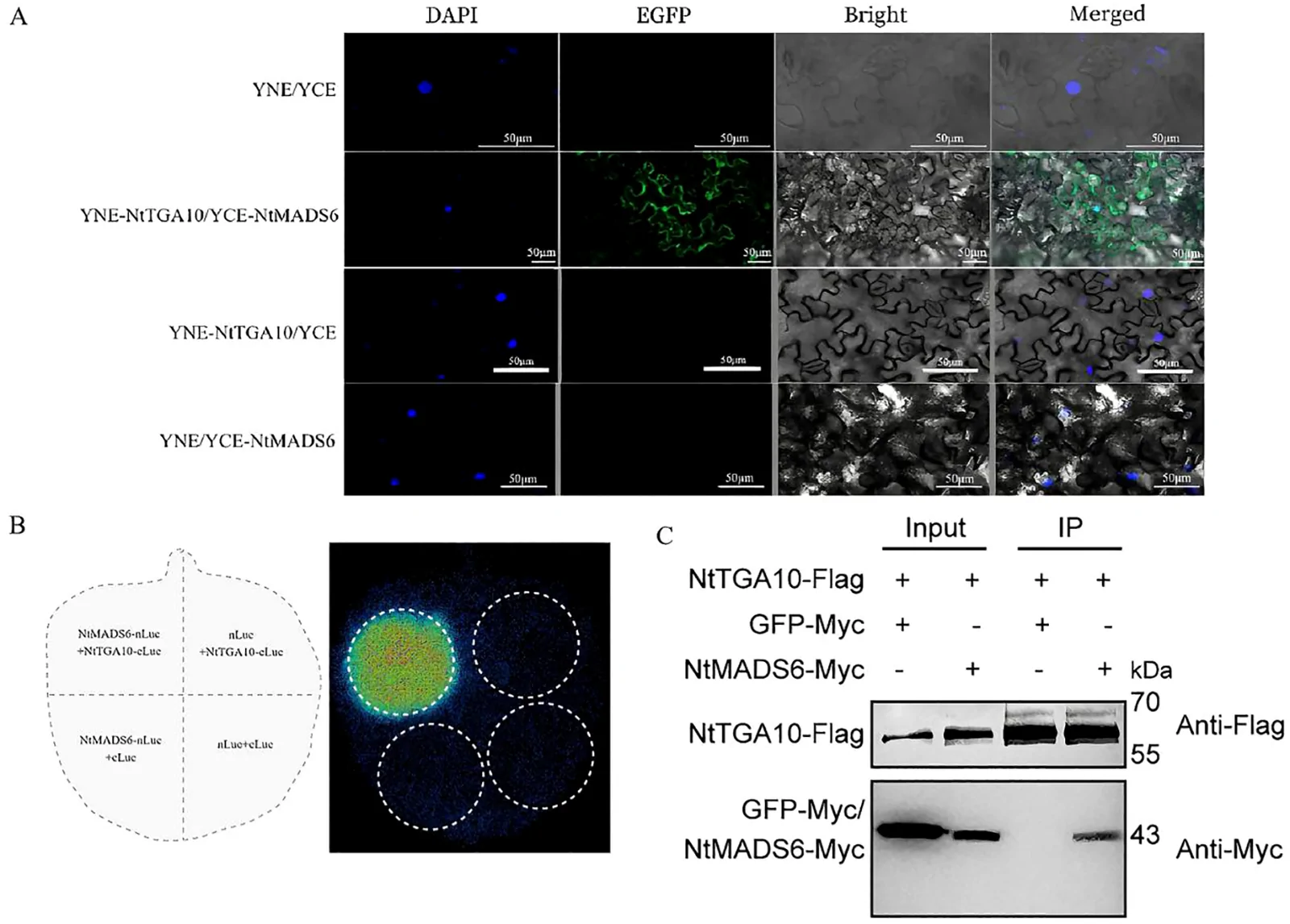
Protein–protein interaction analysis of NtTGA10. **(A)** Validation of the interaction via BiFC. Fluorescence indicates the subcellular location of the reconstituted complex. Blue fluorescence (DAPI) marks the nucleus. The DAPI (blue), GFP (green), bright-field, and merged images are shown in the panels (from left to right). **(B)** Validation of the interaction via LCI. **(C)** Validation of the interaction via Co-IP assay.

In the LCI assay (**Figure 10B**), strong luciferase luminescence signals were detected in *Nicotiana benthamiana* leaves from the experimental group (co-expressing NtMADS6-nLuc and NtTGA10-cLuc). Only background luminescence was detected in the negative control group, confirming the specificity of the interaction signal (co-expressing empty nLuc and cLuc vectors). No obvious luminescence signals were observed in the two negative control groups (NtMADS6-nLuc + cLuc and NtTGA10-cLuc + nLuc).

In the Co-IP assay (**Figure 10C**), NtTGA10-FLAG was detected in the input samples of both control and experimental groups, and was effectively enriched in the post-IP samples. NtMADS6-Myc was detected in the input samples and specifically co-precipitated with NtTGA10-FLAG in the IP samples, whereas the control protein GFP-Myc did not.

### Expression pattern of NtTGA10

The expression pattern of *NtTGA10* was examined in various tissues of JZ and YLL by RT-qPCR. The expression analysis revealed that *NtTGA10* was highly expressed in normal stamens, whereas its expression was significantly downregulated in petaloid stamens (**Figure 11**).

**Figure 11:**
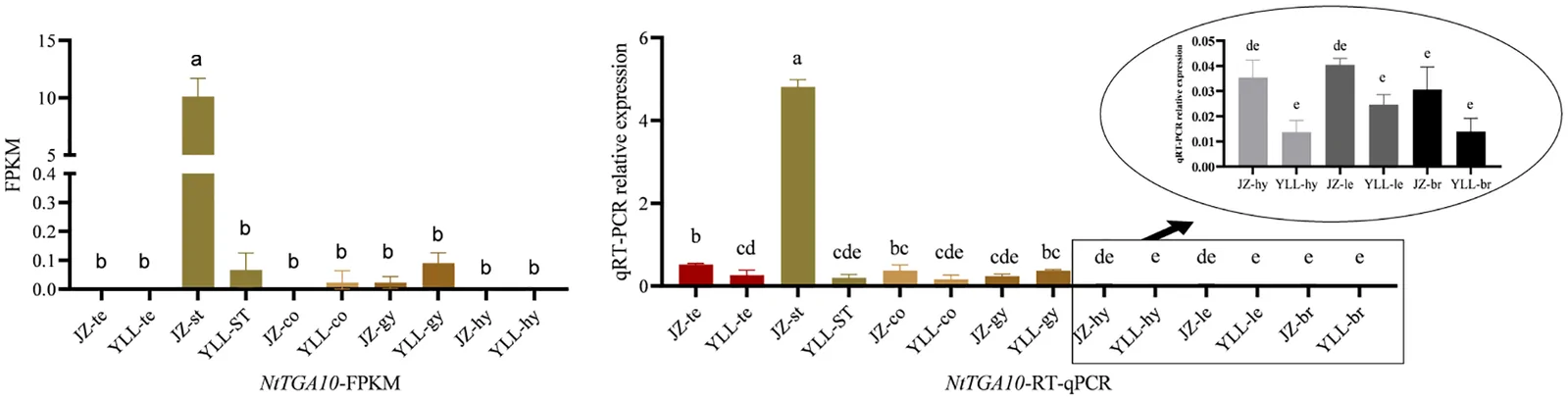
Relative expression levels of NtTGA10 in different tissues. Different lowercase letters (a, b, c, d, and e) above the bars indicate statistically significant differences among tissues (*p* < 0.05).

## Discussion

Staminody (petaloid stamen) is a key developmental event underlying floral diversity and double-flower formation in horticultural plants. However, its molecular regulatory mechanisms, particularly in monocotyledonous ornamentals, remain incompletely understood (Ou et al., 2023). Our detailed anatomical and histological observations firmly establish ‘Yulinglong’ as a natural homeotic mutant of ‘Jinzhanyintai’, in which stamens are converted into petaloid structures while retaining vestigial locule-like internal partitions. This provides a reliable comparative system for investigating the molecular basis of staminody (Ma et al., 2023).

Comparative transcriptomic analysis revealed 231 differentially expressed transcription factors between normal and petaloid stamens across five developmental stages. The enrichment of bHLH, ARF, AP2, and bZIP families corroborates findings in *Alcea rosea* and lily (Gao et al., 2022; Li, 2023; Luo et al., 2024), suggesting a partially conserved regulatory network underlying staminody across angiosperms. Among these, the consistent downregulation of a TGA-subfamily bZIP factor, designated NtTGA10, prompted us to investigate its role.

As important members of the D subfamily of the bZIP family, TGA transcription factors have garnered considerable attention due to their specific binding to TGACG *cis*-acting elements. Previous studies have largely focused on their roles in plant disease resistance and stress responses. For instance, Tian et al. revealed that apple MdTGA2.1 interacts with MdSCL14 to co-regulate salt stress tolerance (Tian, 2013). In addition, the interaction between TGA4 and ERF transcription factors has been demonstrated to participate in the transcriptional regulation of the pathogenesis-related protein gene *PRB-1b* in tobacco (Buttner and Singh, 1997; Van Verk et al., 2009). However, the role of TGA transcription factors in floral organ development has long been overlooked.

Notably, emerging studies have recently begun to uncover critical functions of TGA members in stamen development. In *Arabidopsis thaliana*, *TGA9* and *TGA10* share high sequence similarity and overlapping expression patterns; the *tga9 tga10* double mutant exhibits anther developmental defects similar to those observed in the *roxy1 roxy2* double mutant. Moreover, TGA9/10 directly interact with ROXY1/2, suggesting that glutaredoxins may regulate TGA function through redox modification (Guo, 2023). In tobacco, silencing *TGA2.1* similarly induces the formation of petaloid stamens (Thurow et al., 2005). These findings indicate that TGA transcription factors may be evolutionarily conserved in maintaining stamen identity across different species.

More intriguingly, Sun Mingyue et al. identified a key gene, *PpTGA9*, that links floral development and salt stress tolerance, implying that TGA members might serve as molecular hubs integrating environmental responses and reproductive development (Sun, 2020). Building on this, through transcriptome sequencing and systematic analysis of spatiotemporal expression patterns, we successfully identified the first TGA transcription factor involved in stamen petalody regulation in Chinese narcissus, which we designated *NtTGA10*. Subcellular localization studies have generally shown that TGA transcription factors are localized to the nucleus, as confirmed in plants such as soybean and banana (Guo et al., 2020; Yuan et al., 2024). In the present study, the NtTGA10 protein of Chinese narcissus was also localized to the nucleus, further supporting the high conservation of this localization feature within the family and substantiating its role as a nuclear transcriptional regulator. Together, these findings lay a foundation for elucidating the regulatory mechanism of NtTGA10 in floral organ development.

According to the “ABCDE model” of floral organ development, class C genes play a decisive role in stamen and carpel formation, which has been confirmed in various plants. Among these, *AGAMOUS (AG)* and its homologs are widely recognized as the key regulators of staminody. For example, the loss of *AG* function in *Arabidopsis thaliana* leads to the conversion of stamens into petals (Mizukami and Ma, 1992). Studies in lily (*Lilium*) indicate a close association of the increased staminody in double-flowered cultivars with the downregulation of the class C gene *LelAG* (Akita et al., 2011). Furthermore, staminody in plants such as cyclamen (Tanaka et al., 2013)and camellia (Sun et al., 2014)has also been linked to the aberrant expression of *AG* homologs. These studies collectively suggest that the abnormal expression or function of class C genes, particularly *AG*-like TFs, constitutes a key genetic basis for staminody (Ou et al., 2023). The *AG*-class TF *NtMADS6*, previously identified by the present author group (Chen et al., 2002), was selected to investigate the role of *NtTGA10* in staminody. Also, its protein–protein interaction with NtTGA10 was validated using BiFC, LCI, and Co-IP. These consistent results demonstrated that NtTGA10 interacted with NtMADS6 both *in vitro* and *in vivo*, leading to the hypothesis that they may form a transcriptional complex that participates in the regulation of stamen identity and anther development. However, direct *in vivo* genetic evidence and the identification of downstream target genes of this complex are still required to fully establish its regulatory function.

The present study provides several novel insights that clearly distinguish it from previously published work on TGA transcription factors. First, while TGA factors have been extensively characterized in disease resistance and abiotic stress responses, and more recently in Arabidopsis anther development via the TGA9/10-ROXY1/2 module, our findings establish, for the first time, a direct physical interaction between a TGA protein and a C-class MADS-box protein (AG-like) in the regulation of floral organ identity. This reveals a previously unrecognized transcriptional partnership that links TGA factors to the core ABCDE regulatory network. Second, unlike the Arabidopsis TGA9/10-ROXY module, which operates through redox-dependent regulation, the NtTGA10-NtMADS6 interaction suggests a distinct mechanism, possibly involving direct transcriptional complex formation, to maintain stamen identity. Third, this work is among the first to functionally characterize a TGA factor involved in staminody in a monocotyledonous ornamental species, thereby expanding the evolutionary perspective of TGA function from model plants to horticultural crops. Therefore, the NtTGA10-NtMADS6 module represents a distinct regulatory node in the molecular control of stamen petaloidy, different from previously reported pathways.

In summary, this study establishes a novel NtTGA10-NtMADS6 transcriptional module that is likely involved in maintaining stamen identity in Chinese narcissus. The discovery extends the known function of TGA factors from stress responses to floral organogenesis, and it is plausible that redox or hormonal cues may converge on this complex to modulate reproductive development, although this hypothesis remains to be experimentally tested. Future ChIP-seq or Cleavage Under Targets and Tagmentation (CUT&Tag) experiments will be essential to identify the direct targets of this complex and to reveal the full gene regulatory network underlying petaloid conversion.

We acknowledge several limitations of the current study. First, the functional role of the NtTGA10-NtMADS6 complex in staminody has been validated solely through protein–protein interaction and expression analyses. Definitive genetic evidence, such as overexpression or CRISPR-based knockout of *NtTGA10* in narcissus or a heterologous system, is still lacking and is necessary to establish causality. Second, the direct downstream targets of this transcriptional complex remain uncharacterized; genome-wide identification of binding sites will be crucial to elucidate the regulatory network. Third, narcissus currently lacks a well-established stable genetic transformation system, which precludes in planta functional verification and hinders detailed mechanistic dissection.

Despite these limitations, the identification of *NtTGA10* as a factor whose downregulation correlates closely with petaloid conversion has clear implications for molecular breeding. Targeting *NtTGA10* via gene editing could potentially induce stamen petaloidy and generate male-sterile, double-flowered narcissus varieties without affecting other floral whorls. Such an approach would be highly valuable for the ornamental industry, where double flowers and male sterility are desirable traits. Furthermore, *NtTGA10* and its interacting partner could serve as molecular markers for early selection in breeding programs. However, realizing these applications depends on establishing efficient genetic transformation and gene editing platforms in narcissus, which we advocate as a priority for future research.

## Data Availability

The datasets presented in this study can be found in online repositories. The names of the repository/repositories and accession number(s) can be found in the article/**Supplementary Material**.
