# Correction: Transcriptomic insights into the molecular regulatory mechanism of staminody in Chinese narcissus (*Narcissus tazetta* subsp. *chinensis*)

**DOI:** 10.3389/fpls.2026.2016040

**Published:** 2026-10-08

**Authors:** Pengda Zhao, Yuting Yang, Na Zhang, Keke Fan, Yanting Chang, Baokun Yan, Hanchen Tian, Zehui Jiang, Yanjun Ma, Tao Hu

**Affiliations:** 1 International Center for Bamboo and Rattan, Beijing, China; 2 Key Laboratory of National Forestry and Grassland Administration/Beijing for Bamboo & Rattan Science and Technology, Beijing, China

**Keywords:** *Narcissus tazetta*, protein interaction, staminody, TGA, transcriptional complex

In the **Funding** statement, the funder “Key‑fields Science and Technology Tackling Program of Xinjiang Production and Construction Corps” was erroneously omitted. “This research was funded by ICBR Fundamental Research Funds (grant numbers 1632024017 and 2024AB027)” has been updated to “This research was funded by ICBR Fundamental Research Funds (Grant No. 1632024017) and Key‑fields Science and Technology Tackling Program of Xinjiang Production and Construction Corps (Grant No. 2024AB027)”.

The original version of this article has been updated.

