# Supplementary material for: Transcriptomic insights into the molecular regulatory mechanism of staminody in Chinese narcissus (Narcissus tazetta subsp. chinensis)
Source: Front Plant Sci. 2026 Sep 18;17:1931382. doi: 10.3389/fpls.2026.1931382 (PMC13630926; doi:10.3389/fpls.2026.1931382)
Supplement: Supplementary file 1 [file DataSheet1.docx]

**DNA sequence translation**

*>NtTGA10*

ATGGCAAGTAGAGGGAATAATTCTCAGCATCATCAGCCAGAGAAGCAGATCTCTTTTGGGATGATTCAGCCTAATTCTTCCCCTTCTTCTTCTTCCATGCATGGAAACTTCAACATAACTAAAGAGAGCGGGGCTTATGACTTGGGTGAGTTAGATCAAGCCTTGTTCATGTATCTAGACGGCCAAGATCACTCGTCGGTTCATGAACAACGACAAACTTTGAACATTTTCCCTTCTCAGCCAATGCATGTGGAGCCATCAGCTAAGGTTGCCATGAGTTTGGGTTCTCCAGCAAGCAGTGGAAGCAAGAGAACACCAGGGCCATCCATGGAGTTGGGCAGCACAAGGATTAATGAAGTTCCTACTGCTTCTTTGTCTGGATCCCCCAAGGAAACAAAAGCAATGGTGAAGAAGGAAGGGAACAAAAAGGGAGCGCAATCTAGTTCGGAACATGAAGGGCCCAAGACACCAGATCCTAAGACATTAAGAAGGCTTGCTCAGAATAGGGAGGCAGCTAGGAAAAGCCGGCTCAGAAAGAAGGCTTATGTTCAACAACTTGAGTCCAGCAGGATCAAGCTAAGTCAACTAGAACAAGACCTGCAAAGAGCAAGAGCTCAGGGTGTACTCTTTGGTGGTGGAGCTCTACTTGGTGATCAAGGTCTTCCTGCCGGCATTGGTGGCCTTACCTCAGATGCCGCGGTGTTTGACATGGAGTATGGGAGGTGGTTGGAGGAGCACCACCGACTTATGTGTGAGCTCCGGGCGGCGGTGCAGGAGCACCTGCAAGAGAACGAGTTACGTATGTTTGTGGACAATTGTTTGCACCATTATGATGAGATGTTCAATCTCAAGAGCATGGTTGCAAAGTCCGACGTCTTTCACCTTGTTTCTGGCATGTGGAAGACTCCTGCAGAGCGATGCTTTATGTGGATGGGAGGATTTCGCCCCTCTGAACTAATCAAGATACTCTTGAGCCACATCGAACCATTAACGGAGCAGCAGATACTAGGGATATGTGGATTACAGCAGTCGACGCAAGAGACGGAGGATGCGTTGAGCCAAGGGCTTGAAGCTCTTAACCAATCTCTATCTGATACCGTGGCCTCCGATGCTTTGAGCTGCCCCTCTAACATGGCCAACTACATGGGGCAAATGGCCATTGCAATGAACAAGCTCTCCACCCTCGAAGGTTTTGTTCGACAAGCAGACAATCTGAGGCAACAAACCCTCCACCGACTTCACCAAATCTTGACGACCCGCCAGACTGCCCGGTGTTTCCTGGCCATAGCCGAATACTTCCACCGCCTCCGTGCTCTAAGCTCCCTCTGGCTTGCTAGGCCAAGGCAGGAGTGA

*>NtMADS6*

ATGGGTAGGGGGAAGATAGAGATCAAAAGGATCGAAAACACGACTAATAGGCAAGTCACTTTTTGCAAGCGTCGAAATGGGTTGCTCAAAAAGGCCTATGAATTGTCCGTGCTCTGCGATGCGGAGGTCGCCCTTATCGTCTTCTCTACCCGTGGCCGCCTCTATGAATATGCAAACAACAGTGTGAAAGCGACCATTGAGAGATACAAGAAAGCATGCACTGATACATCCAACACTGCCACTGTCTCTGAGGCTAATTCTCAGTACTACCAACAAGAAGCTTCCAAGTTGCGCCAGCAAATAACCAACTTACAGAATTCTAACAGGAATTTGATGGGGGAGTCTCTGAGCACAATGAGCCTTAGGGACCTGAAGCAGCTTGAGAGCAGGCTAGAGAAAGGCATCAGCAAAATAAGAACTAAAAAGAATGAGTTATTGTTTGCTGAAATTGAATATATGCAAAAAAGGGAGATTGAGTTGCAAAACGATAACATGTACCTACGCAATAAGATAACTGATAATGAGAGAGCACAACAGCAAATGAACATGCTGCCATCAGCTGCTACAACTTCAACTCATGATCAGTACGAGGGGATACCCCAATTTGATTCAAGAAACTTCCTCCAAGTGAGCTTGATGGATCCCGGTCACCACTACTCGCGCCAGCAGCAGACTACCCTTCAACTGGGA

**Accession numbers**

NtMADS6:PZ450893

NtTGA10:PZ450891
