# Supplementary material for: Transcriptomic insights into the molecular regulatory mechanism of staminody in Chinese narcissus (Narcissus tazetta subsp. chinensis)
Source: Front Plant Sci. 2026 Sep 18;17:1931382. doi: 10.3389/fpls.2026.1931382 (PMC13630926; doi:10.3389/fpls.2026.1931382)
Supplement: Supplementary file 2 [file Table1.docx]

Table S1 Primers for real-time PCR analysis

|  | 上游引物（5’-3’）  upstream primer | | 下游引物（5’-3’）  downstream primer |
| --- | --- | --- | --- |
| Isoform0046090 | | GGCTAGTGCTCGCATTCCTAACC | CTGAGTCGTCGGCATCCAAGAAC |
| Isoform0092464 | | GCTGCCATTGCTGACCGTGAA | GCTCTTCCATCTGGTCCAACATTATCT |
| Isoform0081710 | | TCTCTGTTCTTTGTGATACTGATCTCCTAC | ACATAACTTCTCCACCTTCTCAATCTCC |
| Isoform0084718 | | CCAATAGGTGAATCTTCTTCTTCCTCTTCT | CTTGCTGCGGTTGATGTTGTTGAG |
| *NtTGA10* | | AGACATTAAGAAGGCTTGCTCAGAATAGG | AGACATTAAGAAGGCTTGCTCAGAATAGG |
| Isoform0003202 | | AATGGAATGTATAGATACTCGGAGGTTACG | GTTGCTGATGCTGGTGCTGGAA |
| Isoform0045183 | | CCAGAGCAGGCTTAATTGTGAATGT | AGTAGATTTCCCATAACTTCTTCCAGAGG |
| Isoform0072012 | | TGATGAGCAATGGCGGCAGTG | GCTGTGATTGTGGTGGTGAGATAACT |
| *NtTGAL11* | | TTCATCTTATCACTGGAATGTGGACTACC | CGCTCGCAACTGTATCGGCTAAT |
| *Ntactin* | | TGCCCAGAAGTGCTATTCCAG | GTTGACCCACCACTAAGCACAATG |
